# Noncommunicable Disease Prevention and Control in Mongolia: A Policy Analysis

**DOI:** 10.1186/s12889-015-2040-7

**Published:** 2015-07-14

**Authors:** Oyun Chimeddamba, Anna Peeters, Helen L. Walls, Catherine Joyce

**Affiliations:** Department of Epidemiology and Preventive Medicine, School of Public Health and Preventive Medicine, Monash University, Melbourne, Australia; Mongolian Association of Family Medicine Specialists, Ulaanbaatar, Mongolia; Baker IDI Heart and Diabetes Institute, Melbourne, Australia; London School of Hygiene and Tropical Medicine, London, UK; Leverhulme Centre for Integrative Research on Agriculture and Health, London, UK; The Australian National University, Canberra, Australia

**Keywords:** Noncommunicable disease, Health policy, Health planning, Public health, Mongolia

## Abstract

**Background:**

Noncommunicable diseases (NCDs) are the major global cause of morbidity and mortality. In Mongolia, a number of health policies have been developed targeting the prevention and control of noncommunicable diseases. This paper aimed to evaluate the extent to which NCD-related policies introduced in Mongolia align with the World Health Organization (WHO) 2008–2013 Action Plan for the Global Strategy for the Prevention and Control of NCDs.

**Methods:**

We conducted a review of policy documents introduced by the Government of Mongolia from 2000 to 2013. A literature review, internet-based search, and expert consultation identified the policy documents. Information was extracted from the documents using a matrix, mapping each document against the six objectives of the WHO 2008–2013 Action Plan for the Global Strategy for the Prevention and Control of NCDs and five dimensions: data source, aim and objectives of document, coverage of conditions, coverage of risk factors and implementation plan. 45 NCD-related policies were identified.

**Results:**

Prevention and control of the common NCDs and their major risk factors as described by WHO were widely addressed, and policies aligned well with the objectives of the WHO 2008–2013 Action Plan for the Global Strategy for the Prevention and Control of NCDs. Many documents included explicit implementation or monitoring frameworks. It appears that each objective of the WHO 2008–2013 NCD Action Plan was well addressed. Specific areas less well and/or not addressed were chronic respiratory disease, physical activity guidelines and dietary standards.

**Conclusions:**

The Mongolian Government response to the emerging burden of NCDs is a population-based public health approach that includes a national multisectoral framework and integration of NCD prevention and control policies into national health policies. Our findings suggest gaps in addressing chronic respiratory disease, physical activity guidelines, specific food policy actions restricting sales advertising of food products, and a lack of funding specifically supporting NCD research. The neglect of these areas may hamper addressing the NCD burden, and needs immediate action. Future research should explore the effectiveness of national NCD policies and the extent to which the policies are implemented in practice.

## Background

Noncommunicable diseases (NCDs), commonly known as chronic or lifestyle-related diseases are the major global causes of morbidity and mortality. About 63 % (36 million) of the 57 million global deaths in 2008 were due to NCDs [1]. The four major NCDs, i.e. cardiovascular disease (CVD), cancer, chronic respiratory disease and diabetes, account for about 80 % of total NCD deaths and share four common modifiable risk factors: unhealthy diet, physical inactivity, smoking and alcohol consumption [2, 3]. The burden in developing countries is substantial. In 2008, about 80 % of NCD deaths occurred in low- and middle-income countries, up from 40 % in 1990 [4]. NCDs are rising rapidly, are projected to exceed communicable, maternal, perinatal, and nutritional diseases as the most common cause of death by 2030 and to take more lives than all other causes combined if appropriate responses are not taken [1, 5]. Consequently, NCDs result in significant costs to individuals, families, health systems and governments, leading to negative consequences for social and economic prosperity.

Comprehensive, coherent and multi-sector national policies and strategies are required in order to tackle major risk factors leading to the development of and complications associated with NCDs [6, 3]. This has been recognised by the World Health Organization (WHO) 2008–2013 Action Plan for the Global Strategy for the Prevention and Control of NCDs (hereafter referred to as the Action Plan) [7], which supports urgent action to address the rapidly increasing burden of NCDs, especially in low- and middle-income countries. While this has increased awareness of the need for NCD policies, the extent to which it has led to improvements in policy development and implementation, particularly in developing countries, remains unclear [8].

Policy analysis has been widely applied in developed countries, but its application in the health sector of developing countries has been limited [8, 9]. Policy research has been conducted in a considerably higher percentage of high-income countries (72 %) than low- and middle-income countries (LMICs) (57 %) [10]. There is also an acknowledged lack of theoretical and conceptual approaches to analysis of healthy policy in LMICs [11].

Mongolia, the fifth largest country in Asia, and with a population of 3 million, is the least densely populated country in the world. Since the 1990s it has experienced rapid demographic and epidemiological transitions, as in many emerging economies [12]. In Mongolia, CVDs are the leading cause of population mortality [13]. In 2011, CVD accounted for 36.7 % of all deaths, cancer accounted for 20.7 % and external causes of morbidity and mortality for 18.3 %. These causes alone accounted for 75.7 % of total deaths [14].

Since the late 1990s, the Mongolian Government has been building its commitment and reorienting policies to address prevention and control of NCDs. A number of policy documents have been developed to target NCD prevention and control, however, there has been little analysis of the overall suite of policies introduced. The WHO Action Plans provide a guide to developing national policy frameworks and to strengthening surveillance, prevention and management of NCDs and to comparing to its member countries by region. The WHO 2008–2013 NCD Action Plan for the Global Strategy for the Prevention and Control of NCDs has six objectives, with proposed actions recommended for member countries under each objective. This Action Plan was active between 2008–2013 and guided policy development during this time. It has since been followed by the Global Action Plan for the Prevention and Control of NCDs 2013–2020 [15]. An analysis of national NCD policies in countries such as Mongolia, to evaluate the extent to which the objectives of the Action Plan have been met at a national policy level serves several purposes. First, it provides a review of Mongolian progress in this area, at a national level. Secondly, it presents an approach to health policy analysis that others may wish to adopt, important given the paucity of such approaches. Thirdly, it has the potential to identify areas for improvement of the WHO policy framework to better manage the NCD burden globally. In this paper we focus on the alignment between the Action Plan and national policy in Mongolia. The aim of this paper was to evaluate the extent to which policies for NCD prevention and management introduced by the Mongolian Government between 2000–2013 align with the objectives and recommended actions of the Action Plan. Such analysis is important to help identify strengths and weaknesses of the current approach in Mongolia.

## Methods

### Design

A pre-structured case methodology was adopted, with an existing conceptual framework used to define the structure for data collection and analysis [16]. The framework used was the WHO 2008–2013 Action Plan for the Global Strategy for the Prevention and Control of NCDs. More specifically, the proposed actions for member states set out under each of the six objectives of the Action Plan defined the framework used to review Mongolia’s policies and programs. This analysis was undertaken in early 2013. This means therefore that the WHO 2008–2013 Action Plan for the Global Strategy for the Prevention and Control of NCDs was the most up-to-date reference available. (http://whqlibdoc.who.int/publications/2009/9789241597418_eng.pdf).

### Data sources

Health policy is defined as the decisions, plans, and actions undertaken to achieve specific health care goals within a society [17]. A combination of methods was used to identify an initial set of policies for analysis. Documentation relating to relevant Mongolian national government policies, legislation, strategies and programs was collected; 45 policy documents were identified and included in the analysis (Table 1).

**Table 1:** List of all policy documents identified and analysed

Number	
Research and surveys	
1	Global Youth Tobacco Survey 2004
2	Mongolian Steps Survey on the Prevalence of Noncommunicable Disease Risk Factors 2006
3	Mongolian Steps Survey on the Prevalence of Noncommunicable Disease Risk Factors 2009
4	Fourth National Nutrition Survey 2010
5	Multiple Indicator Cluster Survey- 4 2010
6	Salt Intake Survey 2011
7	Global School-based Student Health Survey 2010
8	Global School-based Student Health Survey 2013
9	Health Indicators Yearbook
Policies	
10	MDG- based National Development Integrated Policy 2008–2021
11	Health Sector Strategic Master Plan 2006–2015
12	State Public Health Policy 2001–2015
13	Health Sector Human Resource Development Policy 2010–2014
14	Health Promotion Foundation 2007
15	National Public Health Council 2001
16	Government Policy on Drugs 2002
National programs and strategies	
17	National Programme on Integrated Prevention and Control of Noncommunicable diseases 2006–2013
18	Strategy on Maternal and Child Malnutrition Prevention 2005–2015
19	Health Education Strategy 2010–2015
20	National Food Security Program 2009–2016
21	National strategy on Information, Education and Communication to promote healthy behaviours
22	Updated National Strategy on Information, Education and Communication to promote healthy behaviours 2010–2016
23	National Strategy on Healthy Diet and Physical Activity 2010–2021
24	National Program on Healthy City, District, Workplace and Schools 2012–2016
25	Subnational Programme on Cancer Prevention and Control 2008–2013
26	National Programme on Prevention and Control of Alcoholism 2003–2012
27	Health Financing Strategy 2010–2015
28	Government Platform 2012–2016
29	WHO Country Cooperation Strategy for Mongolia 2010–2015
Guidelines	
30	Clinical guideline on arterial hypertension
31	Clinical guideline on type 2 diabetes
32	Clinical guideline on cervical cancer
33	Clinical guideline on breast cancer
34	“GER” nutrition guideline
Laws	
35	law on Breast Milk Substitute
36	law on Iodine Deficiency Prevention and Iodized Salt
37	Tobacco Control Law
38	Alcohol Control Law
39	Food Law
40	Food Security Law
Others	
41	Millennium Challenge Account–Mongolia Health project 2008–2013
42	Ministry of Health
43	Public Health Institute
44	Centre for Health Development
45	Mongolian Public Health Professionals Association

Relevant documents were identified through searches of websites of the Government of Mongolia, research institutes, United Nations agencies, non–governmental organizations (NGOs), and health professional associations. The key terms used in the website searches were ‘cardiovascular disease’, ‘hypertension’, ‘diabetes’, ‘cancer’, ‘chronic respiratory disease’, ‘asthma’, ‘overweight’, ‘obesity’, ‘nutrition’, ‘physical activity’ combined with ‘policy’, ‘action plan’, ‘strategy’ and ‘guideline’. In addition, general web searches were undertaken using Ovid Medline and Google Scholar search engines to assist in identifying relevant government policies. Key terms included ‘noncommunicable disease’, ‘health policy’, ‘health planning’, and ‘public health’ combined with ‘Mongolia’. Reports and information on national programmes and strategies were also obtained through individual approaches to senior public health professionals in Mongolia. Most documents identified were available electronically and covered the period 2000 to 2013. Policy documents were included if they addressed at least one of the common NCD conditions and their major risk factors, including smoking and alcohol consumption, unhealthy diet, physical inactivity, overweight and obesity, as defined by the WHO [7]. The analysis did not extend to more ‘upstream’ determinants outside the health sector, such as education or transport. A full list is provided in Table 1.

### Data extraction

The first stage of analysis involved creating a matrix to organise and display information extracted from the retrieved policy-related documents. The rows of the matrix comprised the proposed actions for member states under each of the six objectives of the Action Plan. The columns of the matrix comprised the following five dimensions, to obtain key relevant information about each policy document in an objective, consistent manner:

- Data source
- Aim and objectives of document
- Coverage of conditions
- Coverage of risk factors
- Implementation plan or monitoring and evaluation framework

The data source included the title of the particular document and other publication details. The aim and objectives of the document described the overarching goal and specific objectives and strategic priorities to achieve the goal for each document. Coverage of conditions included information regarding which of the NCDs were addressed, including all CVD, hypertension, diabetes mellitus, all cancer and chronic respiratory disease. Coverage of risk factors documented whether common risk factors specified in the Action Plan, such as tobacco and alcohol use, unhealthy diet, physical inactivity, overweight and obesity, salt intake, high blood pressure, cholesterol and blood glucose, were addressed in the documents. The implementation plan dimension was used to document the extent to which each policy included explicit mechanisms or strategies to monitor and evaluate short- and long-term outputs to achieve the goals and objectives. This could include proposed actions, responsible and cooperating agencies, timeframes, monitoring and evaluation stages, expected outcomes, indicators and reporting mechanisms. Information from each policy document was entered into the matrix according to which one or more of the proposed actions it was aligned with. Information about each of the above five dimensions was entered for each policy document.

### Data analysis

Data analysis included data display and confirmation of findings [16]. In the data display phase of analysis, key information identified from each source of the retrieved NCD-related policy documents was entered into the matrix as described above. This data display provided the descriptive information about the policies and programs being undertaken by the Mongolian Government in relation to NCD prevention and control and showed how these aligned with and mapped to the proposed actions under the Action Plan. Many of the documents were in Mongolian, while some were in English. The analyses were undertaken by the first author, who is fluent in both languages. In the second phase of the analysis, patterns and themes were identified. Key documents which were found to be relevant to multiple objectives were identified, and policies and actions under each objective were summarised. Findings were synthesised to draw conclusions about the strengths and limitations of the current Mongolian approach to prevention and control of NCDs. Confirmation of findings involved consulting expert informants (refer to the Acknowledgments for details), reviewing the data display for accuracy and completeness and identifying any additional data sources and references not already included. This resulted in a few adjustments to the descriptions and the inclusion of a few additional data sources. The first author led all phases of the analysis.

## Results

Of the 45 documents analysed, eighteen (40 %) were public health policies, laws and guidelines, thirteen (29 %) were health programs and strategies and fourteen (31 %) were health surveys and documents from government institutes and nongovernmental bodies (Table 1). Although all objectives of the Action Plan were addressed in the included policies, most documents aligned with one or more of the first three objectives. Around half of the policy documents addressed more than one objective (Table 2). The alignment of policies with each objective of the Action Plan from the documents analysed is described below.

**Table 2:** Summary of policy documents against objectives of the Action Plan

	Objective 1	Objective 2	Objective 3	Objective 4	Objective5	Objective 6	Total^a^
	Raising the priority accorded to NCDs	Establishing national NCD policies	Reducing risk factors	Promoting research	Promoting partnerships	Monitoring and evaluating NCDs	
Number of proposed actions	4	13	16	2	2	2	39
Type of policy documents							
Research surveys	4	2	2			8	9
Policies	4	5	1	1	1		7
National programs and strategies	3	4	7		1		13
Guidelines		4	1				5
Laws		4	6				6
Others	1	4		1	2	1	5
Total	12	23	17	2	4	9	45

^a^Total Column does not sum across rows because some documents had multiple counting across objectives

### Objective One: raising the priority accorded to NCDs

This was addressed by 12 policy documents. The National Programme on Integrated Prevention and Control of NCDs 2006–2013 [18], issued by the Government of Mongolia in 2005, is an important document addressing the need for a cross–sectoral response to the NCD burden. As such, this policy highlighted the importance of involving all government sectors, including health, foreign affairs, justice, finance, education, food and agriculture, trade and industry, social welfare, labour, construction and urban development, and defence. The National Programme specified roles and responsibilities of all ministries in implementation of the programme, including specific contributions and commitments towards the achievement of national NCD goals and targets. NGOs and private sector organizations such as mass media, business entities, private industries, and community organizations were also engaged. This document covered prevention and control of major NCDs including CVD, cancer and diabetes mellitus, but not chronic respiratory diseases. Another key document, the Health Sector Strategic Master Plan 2006–2015 [19, 20] addressed all NCDs except chronic respiratory diseases. Each of the four proposed actions within the objective was equally well met.

### Objective two: establishing national NCD policies

This objective was addressed by 23 policy documents and supported at many levels, including key national policy documents, establishment of government-supported health promotion bodies, and clinical guidelines. The key policies aimed to provide a national multisectoral framework to address the NCD burden through involvement of sectors outside of health. Following the release of the State Public Health Policy 2001-2015 [21] a National Public Health Council was established, led by the Prime Minister, and supported by other key ministers. The Government Policy on Drugs established Mongolia’s strategy on drug supply and guaranteed expenses related to the supply of essential drugs by state-owned hospitals to be funded from the state budget. The Drug Policy also encouraged use of generic drugs and stated full or partial subsidization of essential drugs for those living with CVD and diabetes. 2007 saw the establishment of the Health Promotion Foundation [22], which invests in health promotion programs to enforce tobacco and alcohol control, and to prevent tobacco- and alcohol-related diseases. Several documents indicated the existence of dedicated health promotion units within the MOH and other government health authorities responsible for NCDs [10]. Clinical guidelines on arterial hypertension, diabetes, cervical, and breast cancers have been published [23]. The majority of the 13 proposed actions were well met however we were unable to locate clinical guidelines addressing chronic respiratory diseases.

### Objective three: reducing risk factors

This objective was addressed by 17 policy documents and supported at a number of levels, including through legislation, national programs, and strategies. Tobacco control was addressed by the Tobacco Control Law 2005 along with major changes in 2012 that aimed to protect the population from the consequences of tobacco consumption and second-hand smoke [24], in compliance with the WHO Framework Convention on Tobacco Control [25]. The 2012 amendments to the Tobacco Control Law increased excise tax on tobacco by 2 % and banned smoking in all public areas. They also banned the selling of cigarettes to anyone under 21 years old (earlier it was 18 years old). The size of the health warning pictures on cigarette boxes was increased from 33–50 % of the box face. The penalties for violations were also increased. Tobacco advertising – including through movies, the internet, street billboards, posters, and street signs – were also strictly prohibited by the amended Tobacco Control Law. Reducing the harmful use of alcohol was addressed by the Law on Alcohol Control, amended in 2001, 2003, and 2009 [26], and the National Programme on Prevention and Control of Alcoholism 2003–2012 [27]. Food-related policies addressed improving food quality, and supporting healthy diets. The National Strategy on Healthy Diet and Physical Activity 2010–2021 [28] aimed to lower NCD morbidity and mortality by creating an environment for supporting physical activity at individual, community, organization, and national levels. The majority of the 16 proposed actions were well met, except we were unable to identify national guidelines on physical activity and specific food policy actions to address the marketing of foods and beverages to children, as explicitly mentioned in the Action Plan.

### Objective four: promoting research

This was addressed by two policies. The Health Promotion Foundation in Mongolia [22] aims to invest in tobacco- and alcohol-related public health research. There is also a national public health research institution, namely the Public Health Institute, which is the implementing agency of the MOH. Its primary function is to conduct the NCD related surveys, such as the STEPS surveys. There appears to be a lack of a national research funding streams that sustainably support research addressing NCD prevention and control. A series of NCD-related surveys have been undertaken with financial/technical support of international agencies. There has been limited use of these for research purposes, and their main function is monitoring NCDs, as discussed further under Objective 6 below.

### Objective five: promoting partnerships

This was addressed by four policy documents. There was some evidence of building collaborative efforts for NCD prevention and control through partnerships fostered by the United Nations and other international agencies and NGOs. This was reflected in the WHO Country Cooperation Strategy for Mongolia 2010–2015 [29] and the Millennium Challenge Account–Mongolia Health project 2008–2013 [30]. The following example is also illustrative. In 2011, the President of Mongolia initiated Alcohol Free Mongolia and called for a high-level forum for stakeholders to address the harmful use of alcohol with emphasis on creating an appropriate legislative framework to reduce alcohol consumption and its supply. Under the President’s leadership, a national network of 80 governmental and NGOs was established to implement the Alcohol Free Mongolia Initiative [31]. These measures were undertaken as a result of the participation of the President of Mongolia and Minister of Health, with other world leaders, at the 2011 United Nations (UN) High- level meeting on NCDs, which resulted in the government raising the priority of the national NCD burden to high on the development agenda [32].

In addition, as a result of the State Public Health Policy, the National Public Health Council was set up and led by the Prime Minister, with the Vice Minister of Health as secretary and the Vice Ministers of the line and sector ministries of Mongolia appointed as the council members. The council provides strategic guidance and creates linkages to synchronize the implementation of public health programmes [21].

### Objective six: monitoring and evaluating NCDs

This was addressed by nine policy documents. This objective was addressed by the implementation of a series of research surveys: the global youth tobacco survey in 2004 [33], the Mongolian STEPS (Stepwise approach to chronic disease risk factor surveillance) survey on the prevalence of NCD and injury risk factors in 2006 [34] and 2009 [35] using WHO-approved methods, the fourth national nutrition survey conducted in 2010 [36], the multiple indicator cluster survey [37] in 2010, a salt intake survey [38] in 2011, and the global school-based student health survey in 2010 [39] and 2013 [40].

## Discussion

### Summary of the key findings

The Government of Mongolia has led a timely and coordinated response to the emerging burden of NCDs. The multisectoral response has been led from senior levels and based on a population-health approach in tandem with health-systems-strengthening from the primary-care level. Each objective of the Action Plan was generally well addressed by the reviewed policies. Areas not well addressed were chronic respiratory disease, physical activity guidelines and diet-related food standards. There is, as yet, little apparent prioritisation of NCDs in research funding.

### Strengths of the Mongolian approach

The chronology of the identified policies indicates how the each policy was able to build on and complement those already in existence (Table 3). A number of the policy documents strengthened the implementation of the State Public Health Policy [21], such as the Health Sector Strategic Master Plan 2006–2015 [19], and the National Programme on Integrated Prevention and Control of NCDs 2006–2013 [18]. Subsequently, major changes occurred in tobacco and alcohol control laws and a series of nationally-representative surveys on the prevalence of NCD risk factors were implemented. This finding indicates that the NCD-related policies drew on combined or integrated approaches to intervention, and hence, are likely well placed to contribute to reducing the NCD burden. Moreover, the 2011 UN High-level Meeting on NCDs presented a unique opportunity for the Mongolian Government to prioritise a new developmental agenda, exercise leadership and call for action to address NCDs. The Presidential participation at this global meeting demonstrated high level political commitment for NCDs in Mongolia and led to the government undertaking numerous policy measures, particularly, on alcohol and tobacco prevention and control.

**Table 3:** Timeline for issuing of policy documents

	2000	2001	2002	2003	2004	2005	2006	2007	2008	2009	2010	2011	2012	2013
Policies	Alcohol Control Law	State Public Health Policy	Government Policy on Drugs	Law on Iodine Deficiency Prevention and Iodized Salt		Law on Breast Milk Substitute	Health Sector Strategic Master Plan	“GER” Nutrition Guideline	MDG based National Development Integrated Policy	Alcohol Control law^a^	National Strategy on Healthy Diet and Physical Activity	Clinical Guidelines:	Food Law	
													Food Security Law	
												Arterial Hypertension		
						Tobacco Control Law								
		National Public Health Council											Government Platform	
												Diabetes		
											Health Sector HRD Policy			
						Strategy on Maternal and Child Malnutrition Prevention						Breast		
													Tobacco Control law^a^	
												Cervical Cancers		
											WHO CCS for Mongolia			
											Health Education Strategy			
											National Strategy on IEC to Promote Healthy Behaviours			
											Health Financing Strategy			
Program/Strategies				National Programme on Prevention and Control of Alcoholism			National Programme on Integrated Prevention and Control of NCDs	Health Promotion Foundation	Subnational Programme on Cancer Prevention and Control	National Food Security Program			National Program on Healthy City, District, Workplace and Schools	
									MCA-M Health Project					
Surveys					Global Youth Tobacco Survey	STEPS1				STEPS2	Nutrition	Salt Intake		STEPS3
														Global School-based Student Health Survey
											Multiple Indicator Cluster			
											Global School-based Student Health Survey			

*MDG* Millennium Development Goals, *HRD* Human Resource Development, *CCS* Country Cooperation Strategy, *IEC* Information Education Communication, *MCA-M* Millennium Challenge Account-Mongolia, ^a^Major changes, *STEPS* Stepwise approach to chronic disease risk factor surveillance

The findings suggest a comprehensive national multisectoral framework for the prevention and control of NCDs. For example, the State Public Health Policy 2001–2015 [21] resulted in the formation of the National Public Health Council to oversee planning, guidance, monitoring and evaluation of the implementation of national policies with the active involvement of sectors outside of health. Notably, there were strong policy highlights for the importance of involving all government sectors, including health, foreign affairs, justice, finance, education, food and agriculture, trade and industry, social welfare, labour, construction and urban development, and defence. This reflects a whole-of-government and a whole-of-society response, consistent with the Political Declaration of the High-level Meeting of the General Assembly on the Prevention and Control of NCDs in 2012 [20].

The findings also suggest Mongolia, at national policy level, has an integrated approach to addressing NCD-related issues by incorporating multiple conditions and their shared risk factors, and linking these to preventive measures that target the whole population and high-risk individuals. With regards to risk factors, tobacco and alcohol consumption were most commonly addressed, a finding consistent with the findings of the 2010 global survey on assessing national capacity for the prevention and control of NCDs [10]. In a report on the compliance of Asia-Pacific countries with the obligations of the WHO Framework Convention on Tobacco Control, Mongolia was considered to have made good progress towards achieving a healthy tobacco free region [41].

The majority of the documents had implementation plans with clearly formulated monitoring and evaluation frameworks. This aligns with the evidence-based literature which urges that implementation plans should accompany policies, to assist with translating policy into action. When such a plan specifies roles and responsibilities of stakeholders, it provides an important accountability strategy [42, 43].

### Areas for improvement

One notable gap in the Mongolian policies is the failure to address chronic respiratory disease. Mongolia is not alone in this. Many countries (46 %) have policies or strategies for addressing NCDs but neglect to address chronic respiratory diseases [10]. For Mongolia and other countries, it is imperative to incorporate this issue in policy development. Our findings also suggest other gaps in Mongolian policies, including physical activity guidelines, sales or advertising restrictions on food products, and targeted NCD research funding. Hence, we propose that existing national policies and action plans on physical activity and food and nutrition be reinforced with additional strategies so that the importance of the mechanism for engaging physical activity and promoting the responsible marketing of foods and beverages to children is recognised. Prioritised investment in NCD research would also further support and enhance Mongolia’s NCD prevention and control efforts.

### Limitations of the analysis

The scope of the review was limited to publically-available central government policies in relation to the key objectives in the Action Plan [7]. Our analysis did not cover the development, implementation or outcome of the policy, but instead considered the existence of policies as indicators of high-level government commitment and response to NCDs. Hence, we cannot comment on the effectiveness of the overall NCD policies or the extent to which the policies are implemented in practice. Although our analysis is suggestive of a ‘top-down’ approach to policy-making, we have not investigated the development of the policies. Thus, bottom-up and other processes including wider networks and actors [44] may have indeed influenced policy development. Policy analysis is not nearly as neat and rational as this analysis may suggest; policy is not always contained in written documents – decisions can ‘emerge’ in the process of implementation, and inaction is also an aspect of policy making, i.e. what is not done can also be considered policy [9]. The impetus for the Mongolian Government response, and the extent to which it has been influenced by various stakeholders’ groups locally and internationally, therefore remains unknown. This may be an important factor in the sustainability of the policies, and in the success of their implementation.

Having used the Action Plan [7] as the framework against which the Mongolian NCD-related policies were assessed, we are able to offer some comment on the scope of this framework. The proposed actions for member states under the Action Plan were a mixture of broad and specific. Proposed actions must be broad enough to allow WHO member states some flexibility in their response, considering the range of potential strategies and policies and the importance of country context. Recent critiques of NCDs and risk factors prioritised by WHO suggest a more comprehensive approach to addressing NCDs is needed [45]. As NCDs are chronic conditions requiring lifelong management, more comprehensive care is also required. Such care includes primary prevention, early detection, screening, care management, rehabilitation and palliative care at the first contact of service delivery. The need for such care, alongside a focus on the social, economic, and political determinants of disease, has received a stronger focus in the most recent WHO Global NCD Action Plan 2013–2020 [15] which was endorsed by World Health Assembly in May 2013. The WHO Global NCD Action Plan 2013–2020 incorporates the “25x25” strategy, with the goal of a 25 % relative reduction in overall mortality from the four major NCDs by 2025 [46]. To achieve the 25x25 goal, the WHO Global NCD Action Plan 2013–2020 urges the implementation of a comprehensive global monitoring framework, including 25 indicators, and a set of nine voluntary global targets [15]. Based on the current analysis, it appears that Mongolia also incorporated the main components of the objectives of this most recent WHO action plan [15] and the WHO regional action plan on NCDs for the Western Pacific [47]. Nevertheless it will be important to systematically evaluate the implementation of not only Mongolia’s, but also regional and other country initiatives in this new context to ensure progress is made in addressing common risk factors for NCDs.

### Implications

On the basis of our analysis of Mongolia’s policy response to the NCD burden using the Action Plan [7], we conclude that this suite of policies is likely to contribute to and enable the reduction or amelioration of the NCD burden. However, the neglect of chronic respiratory disease, physical activity guidelines, specific food policy actions, and NCD research may hamper addressing Mongolia’s NCD burden. Further action in these areas would enhance current efforts and ensure a comprehensive approach. Recognising the apparent burden of chronic respiratory diseases as one of the leading causes of NCDs globally and in many countries, it is crucial to recognise and incorporate chronic respiratory diseases in the further review and development of national policies in the near future. We believe this analysis provides a useful assessment of the government responses to the NCD burden and illustrates an approach to auditing a policy environment which can be used by other countries. Future research should also explore the effectiveness of national NCD policies and assess the extent to which the policies are implemented in practice, particularly at primary care level.

## Conclusions

This analysis identified a substantial number of Mongolian Governmental policies dedicated to the prevention and control of NCDs, and there was considerable evidence available to suggest that those policies were well aligned with the six objectives of the Action Plan [7]. The Government of Mongolia appears to have successfully generated political commitment, built an appropriate national multisectoral framework for the prevention of NCDs, and integrated the prevention of NCDs into national health policies and undertaken significant endeavours to strengthen the health system at the national policy level.

Despite the majority of the documents reviewed in this analysis having implementation plans with clearly formulated monitoring and evaluation frameworks, it is important to note that it is still unclear to what extent NCD-related policies in Mongolia have been successfully implemented or adequately funded. The mere presence of the NCD-related policies in Mongolia is not indicative that high quality and effective health services are being offered and appropriate preventive activity is being undertaken. The next critical step for Mongolia will be an assessment of the reach and effectiveness of service and programme delivery. Similarly, it is important to continue to strengthen the health system and monitor the progress in addressing NCDs and their risk factors, particularly towards achieving the targets set by the WHO Global NCD Action Plan 2013–2020. This will also assist in determining whether the Mongolian Governmental approach is working to address NCDs and will help inform appropriate policy responses in countries elsewhere.

## Abbreviations

CVD: Cardiovascular disease
LMICs: Low- and middle-income countries
MOH: Ministry of Health
NCD: Noncommunicable disease
NGO: Non–governmental organization
STEPS: Stepwise approach to chronic disease risk factor surveillance
UN: United Nations
WHO: World Health Organisation
